# Vasodilator factors in the systemic and local adaptations to pregnancy

**DOI:** 10.1186/1477-7827-7-79

**Published:** 2009-07-31

**Authors:** Gloria Valdes, Peter Kaufmann, Jenny Corthorn, Rafaela Erices, K Bridget Brosnihan, JaNae Joyner-Grantham

**Affiliations:** 1 Centro Investigaciones Médicas y Departamento Nefrología, Escuela Medicina Pontificia Universidad Católica, Santiago, Chile; 2 Department of Anatomy, School of Medicine, University of Technology, Aachen, Germany; 3 Hypertension and Vascular Research Center, Wake Forest University Health Sciences, Winston-Salem, USA

## Abstract

We postulate that an orchestrated network composed of various vasodilatory systems participates in the systemic and local hemodynamic adaptations in pregnancy. The temporal patterns of increase in the circulating and urinary levels of five vasodilator factors/systems, prostacyclin, nitric oxide, kallikrein, angiotensin-(1–7) and VEGF, in normal pregnant women and animals, as well as the changes observed in preeclamptic pregnancies support their functional role in maintaining normotension by opposing the vasoconstrictor systems. In addition, the expression of these vasodilators in the different trophoblastic subtypes in various species supports their role in the transformation of the uterine arteries. Moreover, their expression in the fetal endothelium and in the syncytiotrophoblast in humans, rats and guinea-pigs, favour their participation in maintaining the uteroplacental circulation. The findings that sustain the functional associations of the various vasodilators, and their participation by endocrine, paracrine and autocrine regulation of the systemic and local vasoactive changes of pregnancy are abundant and compelling. However, further elucidation of the role of the various players is hampered by methodological problems. Among these difficulties is the complexity of the interactions between the different factors, the likelihood that experimental alterations induced in one system may be compensated by the other players of the network, and the possibility that data obtained by manipulating single factors in vitro or in animal studies may be difficult to translate to the human. In addition, the impossibility of sampling the uteroplacental interface along normal pregnancy precludes obtaining longitudinal profiles of the various players. Nevertheless, the possibility of improving maternal blood pressure regulation, trophoblast invasion and uteroplacental flow by enhancing vasodilation (e.g. L-arginine, NO donors, VEGF transfection) deserves unravelling the intricate association of vasoactive factors and the systemic and local adaptations to pregnancy.

## Background

In primates adequate development of the embryo, and later of the fetus, depends on a successful hemomonochorial placentation. This is achieved firstly, by the adaptation of the uterine vessels to pregnancy, with colonization of the uteroplacental arteries by the extravillous trophoblast cells[1,2], and secondly, by the creation of the fetoplacental vascular network of the placental villi from haemangioblastic precursor cells[3,4]. In addition, the systemic maternal circulation adapts to favor uteroplacental perfusion, through increases in plasma volume and cardiac output[5,6].

In this setting the normotension of approximately 90% of human pregnancies, the blood pressure decrement of the second trimester[7], and the reductions in peripheral resistance[8] and sensitivity to angiotensin II[9] are hard to understand. With the belief that an orchestrated conjunction of the various vasodilatory systems participates in the systemic and local hemodynamic adaptations in pregnancy, we have strived to understand their localization, modulation, and potential role. In the following review, a brief description of the main vasodilator systems/agents and their interactions is given, followed by their systemic uterine and placental expression that support their participation in normal pregnancy.

### General aspects of vasodilator systems

#### Prostanoids

**Figure 1:**
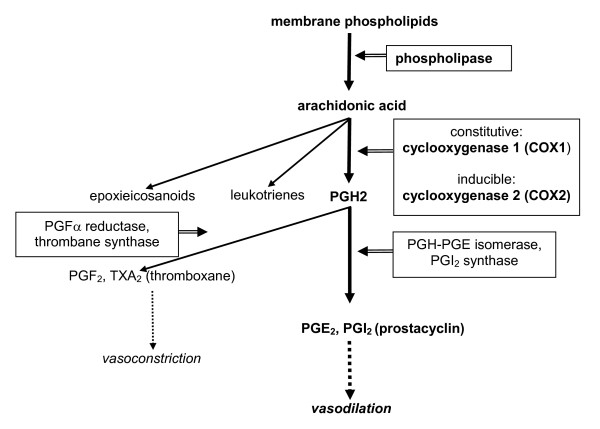
**Synthesis of prostanoids and their respective vasodilator and vasoconstrictor actions**.

Arachidonic acid is an unsaturated constituent of the phospholipid domain of cell membranes. It is mobilized by phospholipases, especially cytoplasmatic phospholipase A2, and is metabolized by constitutive cyclooxygenase (COX-1) and inducible COX-2 into prostaglandins and related compounds, by lipooxygenase into leukotrienes and by p450 into epoxieicosanoid acids[10]. Prostaglandin PGH_2 _is later converted into a variety of eicosanoids by different enzymes. The vasodilators PGE_2 _and prostacyclin (PGI_2_) by PGH-PGE isomerase and prostacyclin synthase respectively. The vasoconstrictors PGF2a and thromboxane (TXA_2_) by PGF2a reductase and thrombane synthase respectively. (Figure 1). The distribution of the enzymes, and hence of the derived prostanoids, is cell-specific.

PGI_2 _is the major vasodilator within the prostaglandin cascade and is synthesized predominantly by the endothelium[11]. Its main effects are mediated either directly, or by opposing the vasoconstrictor and proaggregating effect of platelet-derived TXA_2_. In this review we will focus on PGI_2 _and TXA_2_, as these are the best studied prostanoids in pregnancy.

#### Nitric oxide

**Figure 2:**
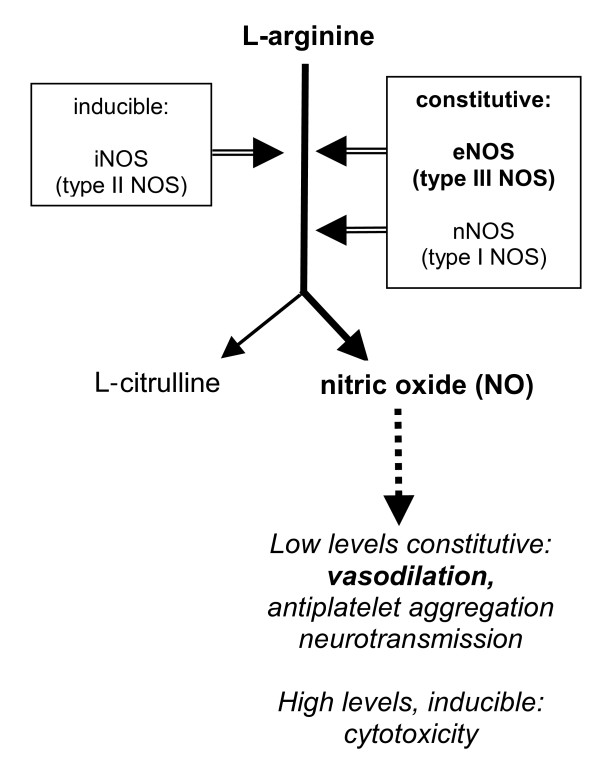
**L-arginine-nitric oxide (NO) pathway indicating the effects of NO according to its generating enzymes and tissue levels**.

Nitric oxide, a potent vasodilator, derives from the oxidation of L-arginine into NO and L-citrulline by nitric oxide synthase (NOS)[12]. There are three cognate forms of NOS, neuronal NOS (nNOS, brain NOS or type I NOS); inducible NOS (iNOS or type II NOS) and endothelial NOS (eNOS or type III) (Figure 2). Endothelial and neuronal NOS are constitutive enzymes, are dependent on Ca^+2 ^and calmodulin, and generate small amounts of NO for vasodilation, maintenance of vascular tone, antiplatelet aggregation and neurotransmission, respectively. In contrast, iNOS is an inflammation-inducible Ca^+2 ^independent enzyme that liberates great amounts of NO. Whether NO plays a protective[13], or a cytotoxic role[14] depends on the magnitude and duration of its synthesis.

#### Kallikrein-kinin system

This endogenous cascade includes a couple of serine proteases, namely tissue and plasma kallikrein, that generate kallidin and bradykinin from precursors, low and high molecular weight kininogens.

**Figure 3:**
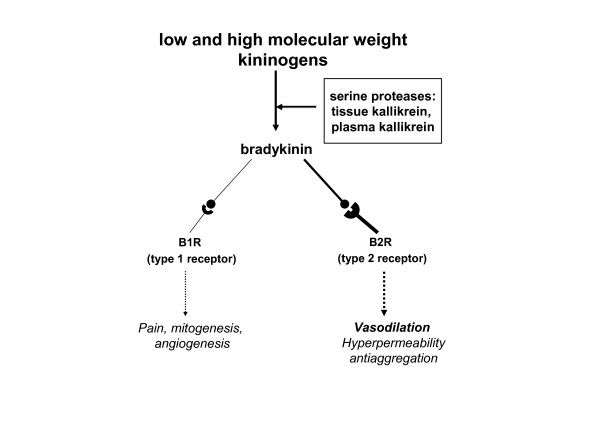
**Kallikrein-kinin system, and the effects of bradykinin according to the type of receptor**.

The effects of kinins, both kallidin and bradykinin are mediated by two types of receptors, type 1 or B1R and type 2 or B2R, acting by way of the G protein coupled receptor (GPCR). By activating the B2R, kinins induce vasodilation, increase vascular permeability and antiplatelet aggregation, both directly or by stimulating the synthesis of nitric oxide (NO) and PGI_2_. On the other hand, acting on the B1R, kinins induce pain and participate in mitogenesis and angiogenesis (Figure 3). [15]

#### Vasodilator components of the renin-angiotensin system

**Figure 4:**
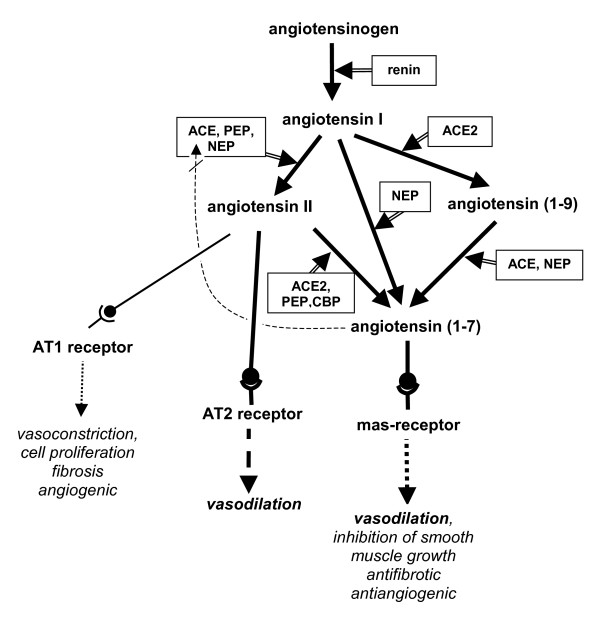
**An updated version of the renin-angiotensin system and the different functions of the active peptides through their receptors**.

Recent findings have added a vasodilator arm to the renin-angiotensin system (RAS), which until recently was only seen as a classical vasoconstrictor system[16,17]. The three components of this vasodilatory arm are angiotensin-(1–7) (Ang-(1–7)) as the vasoactive peptide, Mas, as the respective receptor, and ACE2 (angiotensin converting enzyme 2) as the enzyme linking Ang-(1–7) to the RAS. There are three routes for the conversion into Ang-(1–7). Firstly, it is converted from Ang II (aminoacids 1–8) by cleaving off one amino acid, mediated by ACE2, by prolyl endopeptidase (PEP), or by the carboxypeptidase (CPB). As an alternative, Ang-(1–7) can be directly converted from Ang I (aminoacids 1–10) by cleaving off three aminoacids by neutral endopeptidase (NEP). Finally conversion from Ang I into Ang-(1–7) may take place in two steps, the first mediated by ACE2, which generates Ang (1–9), which is then converted to Ang-(1–7) by ACE and NEP (Figure 4).

Ang-(1–7) binding to the Mas receptor causes vasodilation and inhibition of smooth muscle growth, collagen production, angiogenesis and thrombosis. In addition, the vasodilatory effect of Ang-(1–7) could be partly explained by stimulating NO or PGI2, by potentiating bradykinin via the Mas receptor [18-21], and by competing with Ang I for ACE[22]

In contrast to the classical effects of Ang II mediated by the AT1 receptor (vasoconstriction, cell proliferation, fibrosis, angiogenesis), Ang II binding to the AT2 receptor displays vasodilatory, antiproliferative, antifibrotic and antiangiogenic effects by activating eNOS and kinins [23-25].

**VEGF-A as a vasodilator**: Though there are four endogenous isoforms of vascular endothelial growth factor (VEGF), we refer to VEGF exclusively to VEGF-A. VEGF induces vascular permeability, cell migration and protease production by endothelial cells, all of which are critical components of the angiogenic process, the main function attributed to VEGF[26,27]. In placentation, the modulation of vascular development and remodeling, growth and differentiation by VEGF is shared by placental growth factor (PlGF) and angiopoietins 1 and 2[3,28]; all three of them yield different, but overlapping spectra of activities. In addition VEGF is also an endothelium-dependent vasodilator[29], exerting its effect through NO[30] and PGI_2_[31].

**Figure 5:**
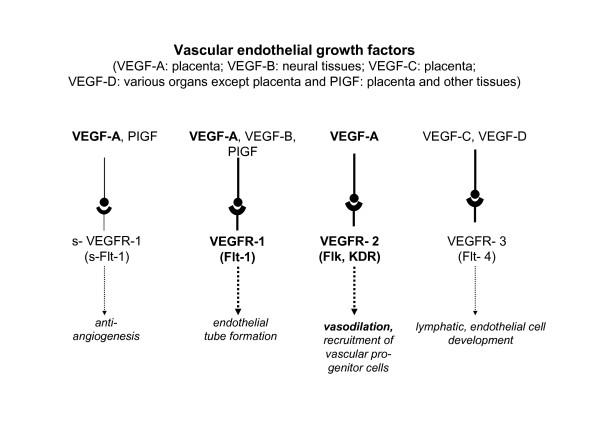
**Different forms of VEGF, their receptors and their respective functions**.

VEGF binds to tyrosine kinase receptors (TKRs), of which VEGFR-1 (tyrosine kinase-1 type fms [Flt-1]), modulated by VEGFR-2 (Flk-1/kinase domain [KDR]), exerts a vasodilatory effect [26,32]. (Figure 5)

**Figure 6:**
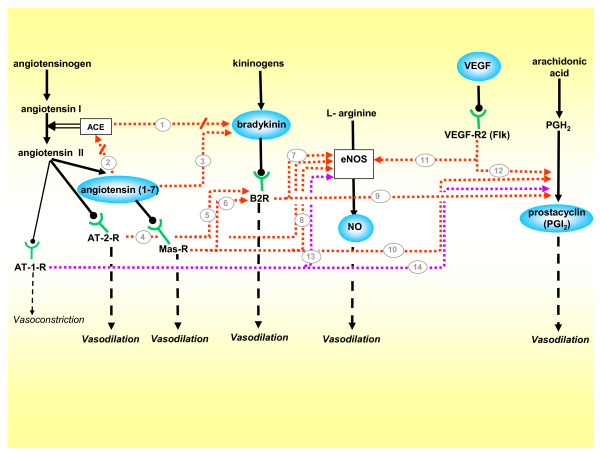
**Vasodilatory network, integrated by the five vasodilators included in this review, and their cognate interactions**. While the majority of pathways are stimulatory, **(1) **ACE degrades bradykinin into inactive peptides, and diminishes its vasodilating effect[13], thus when **(2) **Ang-(1–7) competes with Ang I for ACE it indirectly potentiates bradykinin[16]. **(3) **Ang-(1–7) also enhances bradykinin activity[17]. **(4) **The AT-2-R stimulated by Angiotensin II activates eNOS, and **(5) **the B2R [23-25]. **(6) **The Mas-R activated by angiotensin-(1–7) stimulates the B2R[20], and **(7,8) **jointly with bradykinin activates eNOS[19]**(9,10)**. Activation of the B2R[13] and the Mas-R stimulates prostacyclin synthesis[21]. **(11,12) **The VEGF-R2 stimulates eNOS[23] and prostacyclin production[24]. Finally, the classical vasoconstrictor AT-1-R exerts in gestation a paradoxical vasodilatory effect by **(13,14) **stimulating the synthesis of NO and prostacyclin[95,96]. Stimulatory factors = blue balloons; stimulatory pathways = red arrows; inhibitory pathways = red arrows interrupted by oblique line; pathways exclusive to gestation = purple arrows.

The cognate interactions between the different vasodilator factors are summarized in Figure 6. It is worth mentioning that, as depicted in Figures 1 to 5, these factors have a multiplicity of roles which probably facilitate invasion, placental development and the preservation of an antiaggregated surface of the intervillous space.

### Association of the vasodilator systems to the maternal systemic adaptations to pregnancy

Prostanoids were the first endogenous vasoactive factors studied in pregnancy[33,34], and rapidly prostacyclin and thromboxane became the main protagonists of this system [34-36]. For more than 20 years there has been evidence that in normal pregnancy urinary metabolites of prostacyclin increase progressively, attaining up to 5-fold rise during the last month of pregnancy. As this elevation is not associated to changes in the urinary excretion of thromboxane B2, the balance favors the vasodilator versus the vasoconstrictor balance of this vasoactive pair [37]. In women with severe preeclampsia the urinary excretion of the prostacyclin metabolites is lower than in normotensive women during the last trimester of pregnancy, while thromboxane levels are unchanged, shifting the balance to the debit of the vasoconstrictor and procoagulant TXA_2_. This imbalance, now attributed to an increment in lipid peroxidation and a decrement of scavengers[38], is thought to contribute to the main features of preeclampsia, including hypertension, platelet aggregation, and reduced uteroplacental blood flow. A large scale prospective study shows that the preeclamptic women have lower urinary excretion of prostacyclin metabolites as early as weeks 13 to 16, which yielded a consistently higher thromboxane/PGI2 ratio starting at week 16; however, higher TXA_2 _levels observed after 21 weeks have been interpreted as a secondary event[39].

Following the discovery that prostacyclin and thromboxane levels are disturbed in the maternal circulation in preeclampsia, a number of centers have performed clinical trials with low dose aspirin, believing that treatment with cyclooxygenase inhibitors could prevent or ameliorate the disorder by reducing platelet TXA_2 _production while sparing endothelial PGI_2 _synthesis. Reduction in the incidence of preeclampsia in large scale trials are modest (e.g. 12% in CLASP[40]), suggesting that there is more to preeclampsia than an increment of thromboxane production.

Nitric oxide biosynthesis is increased in pregnant rats, as evidenced by increased plasma and urinary levels of nitrate, and of urinary cGMP[41,42]. In addition, the blockade of NO synthesis in animal models induces marked preeclampsia-like effects[43,44]. Changes of nitrite/nitrate (NOx) levels in different conditions of human pregnancy have been discordant. The discrepancies have been partly attributed to the dependency between dietary intake and plasma and urinary levels; this can be circumvented by a reduction or and control of dietary intake[45]. Sampling under a reduced nitrite/nitrate (NOx) diet has demonstrated a decrease of plasma NOx in the first trimester. This is followed by a later rise to values in nonpregnant women, unchanged urinary NOx, and an increase of urinary cGMP (the second messenger of NO) in the second and third trimester. In preeclampsia plasma NOx and urinary cGMP are similar to normal pregnancy, while urinary NOx is reduced [45-47], so that unequivocal demonstration of a reduced NO synthesis is absent, probably due to lack of systemic or urinary translation of hemodynamically relevant NO production. Nevertheless, elevation of the plasma dimethylarginine (ADMA), an endogenous inhibitor of NOS, in the second trimester is associated with endothelial dysfunction, impaired uterine artery Doppler and the subsequent development of preeclampsia[48].

Valdés et al [50] originally hypothesized that the kallikrein-kinin system in pregnancy could represent a counterregulatory system to the renin-angiotensin system. However, neither the data obtained in the rat or in humans support the view of a contemporary counterregulation. In rats, urinary kallikrein – as a reflection of renal synthesis – rises from gestational day 4 onwards, while plasma renin activity is increased only at day 20, of a 21 to 22 gestation period[49]. In normal pregnant women a rise in urinary kallikrein precedes that of the vasoconstrictors, reaching its maximum between 8 to 12 weeks[50]. Reduced urinary kallikrein levels have been observed in hypertensive pregnancies[51,52], and lower levels predict the preeclamptic syndrome as early as 16 weeks of pregnancy[53].

In normal pregnancy several findings support a predominance of the vasodilator effects of the renin-angiotensin-system, as opposed to an activation of its pressor actions in preeclampsia. Plasma renin activity (PRA) rises progressively along pregnancy, to attain levels 10-fold greater than non-gestational values[7]. In preeclampsia PRA, plasma renin concentration and plasma Ang II are reduced compared to normal pregnancy[52]. Although plasma Ang II concentrations are reduced, women who will develop preeclampsia show a heightened pressor response to Ang II infusion weeks in advance to the clinical manifestation of the syndrome, while this response is blunted in normotensive pregnant women[9]. Wallukat et al. have detected an autoantibody in the serum of preeclamptic patients that binds to the AT_1 _receptor and has agonist activity[54]. AT_1_-B2-receptor heterodimers, which lead to increased Ang II-mediated signalling in smooth-muscle, are highly increased on platelets and omental vessels from preeclamptic patients compared with normotensive pregnant women[55].

The generation of the recently described vasodilator Ang-(1–7) is activated in normal human pregnancy, as demonstrated by a progressive increase of urinary excretion of the peptide starting at 12–14 weeks, and of plasma levels that achieved in late gestation a 1.5-fold rise as compared to non-pregnant values[56,57]. These elevations and the reduced plasma levels of the peptide observed in preeclampsia[57] suggest that this vasodilator exerts a role in the maternal hemodynamic adaptation to pregnancy. In contrast to human pregnancy, in the rat there is no significant change in plasma concentration of Ang (1–7) at the 19th day of pregnancy; however the renal concentration and the urinary excretion increase 5 and 1.6-fold respectively, as compared to virgin animals. In pregnant rats Ang-(1–7) enhanced the dilation of mesenteric vessels, while this effect was absent in the virgin females[58].

Total VEGF circulating levels increase approximately 30 days after embryo transfer, or at week 10 to 14 in spontaneous human pregnancies[59], and continue to rise until 34–36 weeks to a 5-fold increase as compared to postpartum values[60]. These findings suggest that apart from fulfilling an angiogenic effect in reproductive tissues, VEGF may be involved in maternal cardiovascular adaptation to pregnancy. Moreover, a bipolar release of VEGF to the maternal and fetal compartments has been documented in dually perfused human term placental lobules, with predominance of the release to the maternal side[61].

VEGF and plasma from women with preeclampsia induce a concentration-dependent increase in prostacyclin production in bovine endothelial cells, which is inhibited by VEGF antibody[62]. However, binding of VEGF to cell transmembrane receptors is reduced by a soluble circulating form of Flt-1 (sFlt-1), which is generated by alternative splicing. In humans circulating levels of sFlt1 are low in the non pregnant state, high in pregnancy, and extremely high in preeclampsia, probably deriving from an ischemic placenta[63,64]. In this condition most VEGF is bound to the vast excess of circulating sFlt1, and free VEGF levels, which represent active VEGF, are substantially lower than those of total VEGF. In mice, transfection of the sFlt-1 gene generates a preeclampsia-like syndrome, with hypertension, proteinuria, and glomerular endotheliosis, even in the absence of pregnancy or a placenta[63].

The interferences with the activity of VEGF provide a strong support to its vasodilatory role. Moreover, this role is underscored by the effects of the enhancement of VEGF on uteroplacental perfusion. Uterine arteries of pregnant sheep overexpressing VEGF by adenoviral transfection tripled their blood flow in vivo, and in vitro decreased the response to phenylephrine while increasing bradykinin-induced relaxation[65].

**Figure 7:**
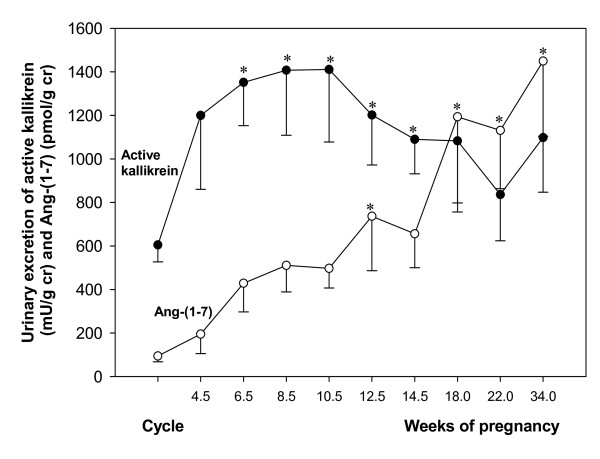
**Urinary excretion of angiotensin-(1–7) and active kallikrein in women in menstrual cycle and in 10 women with normotensive pregnancies**[50,56].

The studies described above support the concept that several interrelated vasodilators are involved in the circulatory changes of pregnancy, probably providing partially redundant systems. It is tempting to speculate that the temporal profiles of the above mentioned vasodilator factors constitute relay systems, which in the changing endocrine milieu of gestation provide constant vasodilation in the mother. This assumption is supported by our finding that the decline in kallikrein throughout pregnancy is associated with a progressive rise of Ang-(1–7) (longitudinal measurements from the urine of 10 normotensive pregnancies[50,66]. (Figure 7)

The functional relevance of maternal vasodilation is underscored by the association of preeclampsia and intra-uterine growth restriction (IUGR) with a reduced plasma volume in spite of elevated aldosterone levels, which precede the clinical onset of either disease[6,67]. A potent vasodilatory effect on the mother is also supported by a marked intragestational reduction of pulmonary artery resistance in a patient with idiopathic pulmonary hypertension, which coincided with elevation of the urinary excretion of vasodilators[68].

### Role of vasodilators in the local adaptation to pregnancy: 1. Animal studies

Three observations lead us to believe that, apart from participating in the systemic maternal hemodynamic changes, vasodilators may play a primordial role in the local uteroplacental adaptation to pregnancy: Firstly, increased vasodilation is observed in the implantation site[69,70]. Secondly, decidual edema to facilitate invasion requires vasodilatory/hyperpermeability factors[71,72] (e.g. VEGF, bradykinin). And finally, vasodilation of utero-placental arteries starts prior to trophoblast invasion of the latter [73-75]. In addition, the respective vasodilatory factors may be involved in regulating fetoplacental perfusion and in protecting the intervillous space and the fetoplacental vessels from platelet aggregation. A series of experiments in animals support this view.

#### Prostanoids

During normal term pregnancy, prostanoid production increases in the uteroplacental unit. Both spectra of prostanoid activity, the vasoconstrictive and pro-aggregatory effects of TXA_2_, countered by the vasodilatory and platelet dissociating activities of prostacyclin serve to maintain a balanced utero-placental circulation. In placenta from salt-loaded pregnant rats TXB_2 _(stable metabolite of TXA_2_) increases, while 6-keto-PGF_1_α (stable metabolite of PGI_2_) decreases[76]. These experimentally induced changes in placental prostanoids are similar to those observed in human preeclampsia (see above); they favor vasoconstriction and are consistent with increased lipid peroxidation.

#### Nitric oxide

The first demonstration of the presence of eNOS in guinea-pig trophoblast was provided by Nanaev and coworkers, who described extravillous trophoblast cells immuno-positive for eNOS[77]. These extend from the subplacenta (the guinea-pig correlate of human cell columns), via the myometrium into the mesometrium; they surround uteroplacental arteries and invade the latter, replacing endothelial cells. This study strongly supports the paracrine role of vasodilators on uterine arteries by demonstrating that the lumen of utero-placental arteries starts dilating already in those segments which are only surrounded by serosal and adventitial eNOS positive extravascular trophoblast cells, but have not yet been invaded. This finding, and the fact that only the already dilated segments were subsequently invaded by trophoblast, challenges the concept that arteries dilate as a consequence of trophoblast-mediated vessel wall destruction (for review see Kaufmann et al. 2003). The data suggest that NO secretion by the periarterial trophoblast constitutes the "pacemaker" that dilates the arteries and turns them into vessels receptive to trophoblast penetration[1].

Also in the guinea-pig, kallikrein, the B2R, eNOS, VEGF and its Flt-1 and KDR receptors, are expressed in the subplacenta, syncytial streamers, and in late pregnancy in the periarterial and intramural trophoblast[78,79]. These data suggest that the vasodilatory effect of NO demonstrated by Nanaev and coworkers may be enhanced by a multifactorial paracrine/autocrine network.

**Figure 8:**
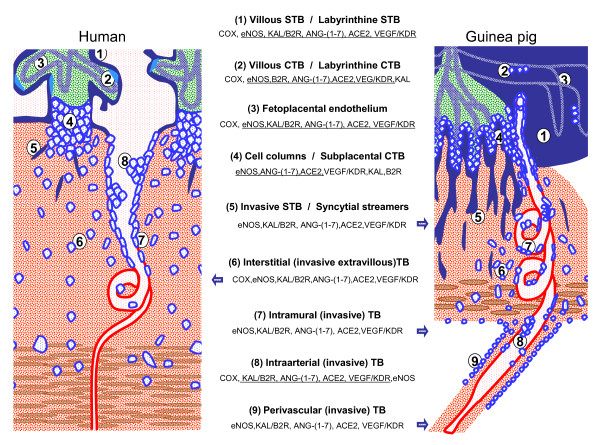
**Schematic representation of the placenta and fetoplacental junctional zone of humans and guinea-pigs**. In order to highlight comparable structures, all fetal tissues are colored blue and green, all maternal structures red and brown. The main cellular and syncytial structures of both placentas are listed together with the reported expression of the vasodilator factors and enzymes. Factors are underlined when present in both species; isolated factors expressed only in one of the two species are lateralized to the presenting species. When the whole panel of factors is described in one of the species, this is depicted by a blue arrow pointing to the respective structures. Note that in the human an equivalent for periarterial trophoblast is not known. TB = trophoblasts; CTB = cytotrophoblasts; STB = syncytiotrophoblast.

The findings in the guinea-pig are of special importance since this species shares with women (a) the hemomonochorial placenta, (b) invasion of the decidua and the uteroplacental arteries by extravillous trophoblast, derived from the subplacenta, the guinea-pig analogue of the human cell columns, and (c) remodeling of the uteroplacental arteries. Moreover, circulating sex steroid levels in guinea-pigs and women follow analogous patterns during pregnancy, and responses to progesterone antagonist treatments are similar[80]. Because of the structural and hormonal similarities, guinea-pigs provide the best non-primate species for understanding the functional role of vasodilators in pregnancy[77,81]. Several morphological and functional homologies between the uteroplacental units of women[82] and guinea-pigs[83,84], and the vasodilator factors expressed in the different cellular subtypes, are summarized in Figure 8.

#### Kallikrein-kinin system

In the rat, kallikrein in luminal and glandular epithelium increases markedly in pseudopregnancy, rises further following intraluminal oil stimulation and in unilateral pregnancy, thus demonstrating its dependency on hormonal and mechanical stimulation [85-87]. A marked increase is observed in the luminal and glandular epithelium of the implantation node, attaining its maximum at day 7, coinciding with the increase in uterine blood flow and permeability. From day 14 onwards kallikrein is absent in the placental labyrinth and trophospongium. The B2R colocalizes with kallikrein, and is also expressed in decidual cells, myometrial and vascular smooth muscle, endothelial cells in decidua and myometrium, and in the fetal endothelial cells of the placental labyrinth[88]. In the light of the findings of Vercruysse et al[89], what we previously defined as retroplacental sinusoids surrounded by cells expressing kallikrein and the B2R, correspond to transformed uterine arteries with perivascular trophoblasts.

#### Vasodilator component of renin-angiotensin system

In the rat, Ang-(1–7) and ACE2 localize in the primary and secondary decidual zone, and in the luminal and glandular epithelium. At gestational day 7 Ang-(1–7) levels are downregulated in the implantation zone. From day 14 onwards Ang-(1–7) and ACE2 are expressed in the labyrinthine placenta, and at day 19 are increased in the uterine wall and expressed in the placenta. In rats submitted to a reduction of uterine perfusion pressure (RUPP), a model that mimics preeclampsia, the placental expression of Ang-(1–7) and ACE2 mRNA is decreased[90]. The dampening of Ang-(1–7) in the implantation zone, its later rise in uterus and the marked placental expression suggest that the peptide is tightly regulated, feasibly to permit the angiogenesis required by placentation, and to enhance perfusion once the placenta is developed. The low levels of Ang-(1–7) and ACE2 in the RUPP model may represent an inhibition of the vasodilator arm of the renin-angiotensin system associated with factors generated by the reduced placental blood flow (sFlt-1, TNF-α, angiotensin type 1 receptor auto-antibody) [91-93]. The uterine and placental concentrations of Ang II are similar to those of Ang-(1–7), but in RUPP animals Ang II predominates over Ang-(1–7). Uterine ACE mRNA increases in RUPP, while it decreases in the placenta[90]. In the guinea-pig Ang-(1–7) and ACE2 are expressed in the labyrinthine and interlobular placenta, in syncytial streamers, interstitial, perivascular and intravascular cytotrophoblasts; in contrast to other vasodilator factors, no expression of Ang-(1–7) and ACE2 have been detected in the subplacenta, providing evidence of a tight regulation of the peptide and its generating enzyme (Joyner, Brosnihan, Corthorn, unpublished observations),

Surprisingly, in pregnant sheep the infusion of Ang II evokes a decreased vascular resistance, associated with stimulation of NO and PGI_2 _production[94,95]. The ovine placenta expresses both angiotensin receptors, with predominance of the AT1-R in early pregnancy, and similar expressions of the AT1 and the AT2-R in late pregnancy. The AT1-R and eNOS colocalize in the fetoplacental endothelial cells, and the AT1-R has been shown to mediate the Ang II increases in eNOS protein expression and NO production[96]. In turn, NO can stimulate angiogenesis and attenuate Ang II vasoconstriction. The paradox that the potent vasoconstrictor Ang II exerts a vasodilatory effect via eNOS and PGI_2 _in the uteroplacental unit further stresses the physiological relevance of vasodilation in pregnancy. These responses are unique to pregnancy, and to the endothelium of uterine arteries, as they have not been observed in virgin sheeps and in systemic arteries of pregnant ewes [97]. The important role of the vasodilatory effects of Ang II in pregnancy is emphasized by the fact that these are absent in the uterine arteries of the non-pregnant sheep and in systemic arterial segments from pregnant animals; furthermore, it is underlined by the catastrophic effects on fetal hemodynamics of either converting enzyme inhibitors or by blockade of the Ang II type 1 receptor [98-101].

### Role of vasodilators in the local adaptation to pregnancy: 2. Studies in human reproductive tissues

The data obtained in animal experiments are supported by several descriptive findings and *in vitro *studies on human reproductive tissues.

#### Prostanoids

Trophoblast cells, endothelial cells, macrophages, fibroblasts, smooth muscle and decidual cell express cyclooxygenases; COX-2 is co-expressed in macrophages, fibroblasts, endothelial cells and smooth muscle cells. COX-1 mRNA expression does not differ in placenta of preeclamptic pregnancies, but is elevated in the placental bed, as compared to normal pregnancies; while COX-2 mRNA was unchanged. The thromboxane/prostacyclin ratio, as well as lipid peroxides, are higher in villous cytotrophoblast, and villous core from preeclamptic pregnancies than in those of normal pregnancies[102]. These results, which agree with earlier work on placental tissue[103], indicate that the placenta, and in particular the cytotrophoblast, may contribute to the imbalance of thromboxane and prostacyclin observed in preeclampsia, which likely promotes the coagulopathy and heightened vascular responsiveness so characteristic of this condition.

#### Nitric oxide

NOS activity has been described in human trophoblast from early placenta (first trimester), term placenta and myometrium. In the first trimester immunohistochemistry demonstrates eNOS in the villous syncytiotrophoblast, the cell columns of anchoring villi and in extravillous trophoblast at the implantation site. It is conceivable that relaxation of the vascular walls at the implantation site may be caused by NO provided by trophoblast cells. As described above for the guinea-pig placenta, NO and related local vasodilators may well be involved in the maximum dilation of uteroplacental arteries. This process was first described and attributed to trophoblast-derived wall destruction by Robertson and coworkers[104]. It starts at 8 weeks of gestation[105], and thus before onset of arterial wall decomposition by invasive trophoblast cells[74]. Only the already dilated arteries are subsequently invaded by trophoblast. Lyall and coworkers[106] could not find NOS expression in invasive trophoblast in human placental bed biopsies. This negative finding was, however, contradicted by Martin and Conrad who found immunohistochemical and in-situ-hybridization proof for the extravillous trophoblastic expression of eNOS[107]. Taken together, it is meanwhile quite likely that the classical hypothesis of uteroplacental arterial dilatation as a consequence of trophoblast-induced wall destruction must be revised and that the NO-induced dilatation described for the guinea-pig by Nanaev and coworkers can be transferred also to the human condition (for review see Kaufmann, Black, Huppertz 2003)[1].

Significantly higher and undisputed activities of eNOS were found in villous trophoblast, suggesting a relation between number of trophoblastic cells and eNOS activity[108]. NO formation in villous syncytiotrophoblast may importantly contribute to dilatation of underlying fetal vasculature, and may prevent platelet aggregation when released into the intervillous space. The role of placental NO is backed by several functional studies. In isolated perfused human placental cotyledons from normal and preeclamptic pregnancies, blockade of NO synthesis, but not indomethacin, increases the resting fetal perfusion pressure; 5-hydroxytryptamine causes greater increases in perfusion pressure in preeclamptic than in normal placentas. In chorionic arteries and veins from normal placentas, NO blockade enhances the pressor effect of 5-hydroxytryptamine; in preeclamptic placentas this effect – which is reduced by COX inhibition – is not significantly modified[109]. These results suggest that basal release of NO, but not of vasodilator prostanoids, may contribute to the low resting vascular tone in normal placentas, and to attenuate the strong vasoconstrictor effect induced by 5-HT.

In dually perfused placental cotyledons several NO inhibitors (N omega-nitro-L-arginine (NOLA), hemoglobin and methylene blue) increase fetal vessel basal perfusion pressure and also increase the constriction induced by the thromboxane mimetic U46619[110]. NOLA markedly potentiates the constrictor effects of endothelin-1, angiotensin II, and 5-hydroxytryptamine showing that NO contributes to maintain low basal fetal vessel impedance, and reduces the effects of vasoconstrictors; thus reduced NOS activity could contribute to the pathogenesis and/or effects of preeclampsia. In contrast with the changes associated with NO, COX inhibitors do not affect fetal vessel basal perfusion pressure nor potentiate the effects of the thromboxane mimetic.

In perfused segments of human umbilical artery and vein with intact endothelium the relative release of PGI_2 _and NO has been compared, utilizing the cascade bioassay. The basal release of NO from the artery is approximately five times greater than that of PGI_2_. After stimulation with the calcium ionophore A-23187, the release of NO from vein and artery increases five to six-fold, and is three times greater compared with that of PGI_2_. NO is also more potent in relaxing endothelium-denuded fetoplacental vessels *in vitro *relative to PGI_2_[111]. These studies suggest that NO is more important than PGI_2 _for maintenance of low vascular tone in fetoplacental vessels.

The importance of NO is also underscored by the improvement in the local and systemic perfusion changes that accompany the supplementation of hypertensive pregnant patients with L-arginine, the substrate of NOS. Supplemented patients show reductions of arterial pressure and the resistance of uterine arteries both in the short and long term[112,113]; in the long term L-arginine supplementation is also associated with improvement in blood pressure control and NO synthesis [113-115]. These benefits pose the need to extend the preliminary studies with L-arginine supplementation and NO donors.

The evaluation of the local expression of eNOS in preeclampsia has yielded discordant results. Myatt et al. comparing the presence and localization of eNOS and nitrotyrosine (a marker of peroxynitrite formed by interaction of NO and superoxide) in placental villi from normotensive pregnancies and pregnancies complicated by preeclampsia, found that the later displays greater nitrotyrosine immunostaining in vascular endothelium, in the surrounding vascular smooth muscles and in villous stroma[116]. Moreover, they found intense eNOS staining in endothelium of stem villous vessels and the small muscular arteries of the terminal villi. Consistent with these findings Shaamash et al. found that both placental NOS activity and NO end-products (nitrites and nitrates) are significantly higher in villous homogenate of preeclamptic placentae compared to those of normal pregnancy; in addition, the increased NOS activity and NO production are directly related to the severity of this syndrome[117]. Recently, Norris et al. found that nitric oxide metabolites (nitrites) in the uterine vein draining the placenta in preeclamptic women delivered by cesarean section are higher than in normotensive pregnancies [118]. Based on these findings, Myatt, Shaamash and Norris postulate that increased NO placental production represents a compensatory mechanism to offset the pathologic effects of preeclampsia.

Schiessl on the other hand, found no differences in immunohistochemical expression of eNOS in the syncytiotrophoblast and extravillous trophoblast of placentas of normal controls and preeclamptic mothers, although placentas with IUGR show a decreased immunoreactivity and eNOS protein content, as estimated by Western blotting[119]. Work by our group demonstrates that eNOS is similarly expressed in the syncytiotrophoblast and in the fetal endothelium in control and preeclamptic pregnancies[120], coinciding with the findings of Orange et al., who in this light, have excluded a possible pathogenic role for eNOS in this disease [121]. Wang et al., found a similar nitric oxide production in placental homogenates from preeclamptic and normal term pregnancies[122]. In addition, Conrad and Davis, measuring the conversion of [^3^H]arginine to [^3^H]citrulline, found that NOS activity in the villi of placentae from women with preeclampsia is not significantly different from normal pregnancies [123].

Taking a different stance, Noris et al. have shown that the gene expression and protein content of arginase II – an enzyme that degrades arginine to ornithine- are higher in preeclamptic villi than in normotensive pregnancy. Moreover, in this study the concentration of the NO precursor L-arginine in umbilical blood and in villous tissue is lower in preeclampsia than in normotensive pregnancy. They postulate that the lower normal L-arginine concentration caused by arginase II overexpression redirects eNOS toward peroxynitrite[124].

#### Kallikrein-kinin system

In samples obtained from sporadic miscarriages, as well as from early and late third trimester, kallikrein mRNA is present in syncytiotrophoblast, cytotrophoblast, and fetal endothelial cells of placental villi, in decidual cells, in the basal and chorionic plate vessels, in trophoblastic cell columns, in interstitial and intraarterial trophoblast cells. Tissue kallikrein protein is present in the same cell types with the exception of trophoblastic cell columns and the various types of extravillous trophoblast cells[120]. In first trimester syncytiotrophoblast the intensity of the signal is higher than that observed in normal term placentas. The immunoreactivity of the B2R shares the same distribution of kallikrein mRNA, and like kallikrein, is more intense in the first trimester as compared to term. In placenta accreta, a condition of exaggerated invasiveness, the expression of kallikrein is increased in syncytiotrophoblast, and that of the B2R in the fetal endothelium and in extravillous trophoblast; in contrast to normal pregnancy, kallikrein is expressed in extravillous trophoblast. In preeclampsia, the only observed difference is an increased B2R signal in the extravillous trophoblast. Because of the intense mixture of different cell types composing the different uteroplacental zone, and the inability to separate them, the variations within the various cells types have not been confirmed by western blotting.

Amarnani et al. perfused isolated human placental cotyledons with bradykinin at low concentrations and produced a concentration-dependent decrease in perfusion pressure, whereas higher concentrations increased perfusion pressure[125]. The decreased perfusion pressure provoked by low dose bradykinin is potentiated by captopril (which blocks bradykinin degradation) and is attenuated with the B2-receptor antagonist HOE-140 or by pretreatment with an inhibitor of nitric oxide synthase. On the other hand, a COX inhibitor shows no effect. The latter two results underline the importance of the vasodilatory effect which bradykinin has on eNOS activity, but they question the respective effect on prostacyclin production (cf. chapter on General Aspects of Vasodilatory Systems: Kallikrein-Kinin System).

#### Vasodilator components within the renin-angiotensin system

The expression of renin in placenta, uterus and fetal membranes has been reported since the late 60s [126-128]. The functional importance of the renin-angiotensin system is illustrated by several facts: by the severe neonatal complications observed in women treated with converting enzyme (ACE) inhibitors[129,130]; by the association of preeclampsia to autoantibodies to the Ang II type 1 receptor, and by the association of preeclampsia to the M235T polymorphism of the gene that codes for angiotensinogen[131,132]. The important role RAS plays in pregnancy is further underlined by the fact that a preeclampsia-like model has been generated in female mice transfected with the human angiotensinogen gene and mated with males transfected with the human renin gene underlines this role[133]. The novel vasodilatory components of the renin-angiotensin system, Ang-(1–7) and its generating enzyme ACE2 are expressed in syncytio and cytotrophoblast, endothelial cells, vascular smooth muscle of placental villi, interstitial and intraarterial trophoblast, in decidual cells, and in the endothelium and smooth muscle of the umbilical arteries. As for kallikrein, the immunoreactivity for the peptide and enzyme are greater in first trimester samples[134]. With the exception of an increased expression of ACE2 in umbilical arterial endothelium in preeclampsia, the expression of Ang-(1–7) and ACE2 is similar in different cell types in reproductive tissues from normal term or preeclamptic pregnancies. However, Ang-(1–7) does not change in the chorionic villi of preeclamptic women, whereas Ang II is increased; this suggests that the balance of these two biologically active peptides may be shifted toward the Ang II vasoconstrictor arm of the RAS[135]. The differences in tissue versus circulating concentrations of the various components of the RAS are highlighted by the fact that Ang-(1–7) is decreased in the blood of preeclamptic women[57].

Messenger RNAs of renin, angiotensinogen. ACE, ACE2, NEP, AT1, AT2 and Mas receptors have been demonstrated in placenta and placental bed[136]. Immunoreactivities for angiotensinogen and AT1R are increased in chorionic villi and decidua of preeclamptic subjects, as compared to normal[135]. Herse et al., has found an upregulation of decidual AT1R mRNA in preeclampsia; in contrast, the vasodilator AT2 receptor gene expression is present in 4% of the preeclamptic versus 60% of controls[135,137].

The assumption of an interaction between bradykinin and the renin-angiotensin system is supported by the observation that Ang I at concentrations present in the fetal circulation, but not Ang II, potentiates the vasodilator effect of bradykinin in the fetoplacental vessels[125]. This enhancement may relate to Ang-(1–7) which also derives from Ang I.

#### VEGF

Is expressed in the non-pregnant endometrium, and its transmembranous receptors VEGFR-1 (Flt-1) and VEGFR-2 (KDR), as well as s-VEGFR-1 (sFlt1-1), display a cyclic regulation throughout the menstrual cycle[138,139]. VEGF seems to be crucial for uterine receptivity, as its mRNA expression is detected in the blastocyst; its translation may enable the implanting ovum to induce angiogenesis – and probably hyperpermeability – at the implantation site by binding to the ligand of endometrial receptors[140]. Later in pregnancy VEGF is expressed mainly in villous cytotrophoblasts[141], in syncytiotrophoblast[142], the invading front of trophoblastic cell columns, extravillous[143] and in endovascular cytotrophoblast. VEGF-receptor binding stimulates the cytotrophoblastic expression of integrin α1, a molecule of the invasive repertoire that interacts with the maternal extracellular matrix and is characteristic for the invasive subset of extravillous trophoblast[144]. At early gestational stages, immunoreactivities of VEGF-C, VEGF-R1 and VEGF-R2 in intramural extravillous trophoblast (EVT) are reduced as compared to those in mononuclear interstitial and intraarterial EVT. The intramural EVT expression for VEGF-C increases along pregnancy, while a reduction in VEGF-R1 and VEGF-R2 by intraarterial EVT was observed[145]. VEGF in perivascular trophoblast could through its vasodilator effect prime the uterine arteries for invasion, as suggested for NO[77], thus enhancing their high blood flow.

As already mentioned earlier, VEGF exerts its vasodilatory effects via activation of eNOS and of prostacyclin synthesis. This has been shown also for the human placenta: Dual perfusion of human term placental lobules with VEGF at physiological concentrations exerts a potent vasodilation of the fetoplacental vasculature, partially mediated by VEGFR-2, via NO as a second messenger[61].

## Conclusion

**Figure 9:**
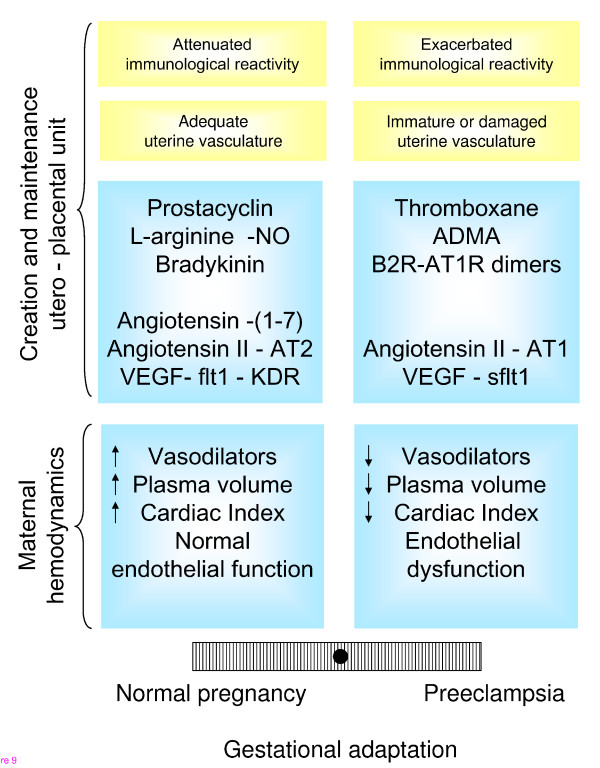
**Balance between immunological, morphological and vasoactive factors that determine an adequate or a defective adaptation to pregnancy, both at the level of the uteroplacental unit and the mother**. For the purpose of this review emphasis has been given to the local and systemic hemodynamic adaptations.

The data listed above demonstrate that at least five different vasodilator systems are involved in the control of uteroplacental and fetoplacental hemodynamics throughout pregnancy, the vasodilator component of the renin-angiotensin system, the kallikrein-kinin system, prostacyclin, nitric oxide and VEGF. Several decades after Walsh proposed a balance between the vasoconstrictor thromboxane and the vasodilating prostacyclin to sustain normal pregnancy, the number of players in this delicate balance has expanded; furthermore the local balance has to be extended to that of maternal hemodynamics, as proposed by Wallenburg [146-148] (Figure 9). The multiplicity of components that integrate the vasodilator surge of pregnancy probably exerts compensatory functions and provides finely tuned relay systems in the changes in the hormonal milieu of pregnancy, and in the oxygen tension of the intervillous space. Moreover, there is increasing evidence that the various vasodilator systems intensely interact, partly only modulating each other, partly even using parts of the other systems to exert their own function, e.g. VEGF stimulating eNOS activity and prostacyclin production. Some of these interactions seem to be specific for pregnancy, e.g. upregulation of prostacyclin by Ang II.

There are still uncertainties, contradictions and data, e.g.eNOS/NO, that seemingly do not fit into the picture of a functioning vasodilator network. This is mainly due to the fact that a more detailed analysis of the data is hampered by various problems: The environment, diet and genetics of the individuals studied. The estimation of activities of the involved factors, often based on semiquantification of immunoreactivities, Northern and Western blotting, must be generally interpreted with caution. The impossibility of early sampling in pregnancies that can be characterized as normal or preeclamptic at term, the restricted availability of normal human material from the second trimester, and the difficulties inherent to placental bed biopsies even at term are further obstacles when attempting to interpret longitudinal profiles of the various players. By contrast their circulating levels and urinary excretion data are reliable, but difficult to interpret because of the huge variety of potential sources of secretion/excretion. Finally, having in mind the complexity of the vasodilator network, *in vitro *studies on human tissues and experimental manipulation of single factors in animal studies do not only bear the danger that data may be difficult to translate to the human; moreover, it would not be surprising if experimental alterations induced in one system are compensated by the interacting with other players of the network. The same, of course, is valid when studying serum or urine data from patients from pregnancy disorders with or without treatment.

In spite of these caveats, the data provided by many groups suggest that preeclampsia is a typical example of a disorder in which the balance between vasoconstrictor and vasodilator systems, and possibly even the balance within the vasodilator network, is disturbed (Figure 8). This by no means implies that a deranged vasoconstrictor/vasodilator balance is sufficient to explain the pathogenesis of preeclampsia. It is clear that cell biological interactions between invading trophoblast and uteroplacental arterial walls, general problems of the uterine or even systemic vasculature of the mother, as well as the immunological interaction between mother and her semiallograft, provide alternative underlying pathogenetic mechanisms.

## Competing interests

The authors declare that they have no competing interests.

## Authors' contributions

GV, JC and RE drafted the manuscript; PK and GV conceived this review and jointly with KBB did major contributions to the themes dealt in it; JJG performed immunohistochemical studies. All authors read and approved the final manuscript.

## Authors' informations

PK is a placental morphologist and pathologist, who has coauthored the five editions of Pathology of the human placenta (eds. Benirschke K, Kaufmann P, Baergen RN. Pathology of the human placenta. New York:Springer), KBB and JC have contributed to the findings of the novel vasodilatory arm of the renin-angiotensin system and to the study of the kallikrein-kinin system respectively, and GV combines the management of hypertensive pregnant women with research in vasoactive factors in pregnancy.
